# Pandemic Dynamics and the Breakdown of Herd Immunity

**DOI:** 10.1371/journal.pone.0009565

**Published:** 2010-03-15

**Authors:** Guy Katriel, Lewi Stone

**Affiliations:** Biomathematics Unit, Faculty of Life Sciences, Tel-Aviv University, Tel-Aviv, Israel; University of Bristol, United Kingdom

## Abstract

In this note we discuss the issues involved in attempting to model pandemic dynamics. More specifically, we show how it may be possible to make projections for the ongoing H1N1 pandemic as extrapolated from knowledge of seasonal influenza. We derive first-approximation parameter estimates for the SIR model to describe seasonal influenza, and then explore the implications of the existing classical epidemiological theory for the case of a pandemic virus. In particular, we note the dramatic nonlinear increase in attack rate as a function of the percentage of susceptibles initially present in the population. This has severe consequences for the pandemic, given the general lack of immunity in the global population.

## Introduction

The renewed interest in modelling the dynamics and forecasting the evolution of emerging and reemerging diseases has been spurred on further with the arrival of the recent new H1N1 influenza pandemic [1], [2]. Unfortunately, conventional modelling techniques are usually, at best, only able to provide a general idea of how a pandemic might evolve, since crucial information concerning model parameters is generally unavailable. For example,the initial fraction of susceptibles in the population 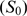, and the basic reproductive number 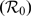 are rarely known, and are often difficult to estimate. Yet without accurate information on both of these, it is impossible to predict the proportion of the population infected, or attack rate, of the the epidemic.

To make the problem more transparent, during the start of the recent H1N1 pandemic, there were several large-scale research efforts into estimating the reproductive number 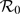 of the new influenza strain in a fully susceptible population. However, from the initial growth rate of the epidemic one can only estimate the effective reproduction number 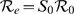 and *not* the two parameters 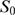 and 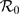 separately. The equality 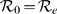 holds only if 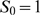 (the entire population is susceptible), which may not be the case for past pandemics and the new influenza strain H1N1 [3]. The importance of distinguishing between the basic reproductive number 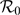 and the effective reproductive number 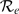 is far from a merely terminological matter. For example, consider two epidemics, one with 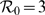, 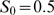 and the other with 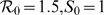. In both cases 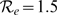, and the two epidemics will initially grow at the same exponential rate, but the final attack rate of the epidemic with 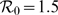, 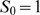 will be *twice* that of the epidemic with 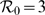, 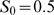. Thus measuring growth rate at the beginning of an epidemic cannot provide one with a prediction for the future, unless one has an independent estimate of the fraction of susceptibles 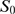 [4], [5].

In this paper we discuss an approach to partially address these problems, to estimate key variables and to make projections. Our methodology is general, but we focus on the specific case study of influenza, because of the exigency of the current pandemic and because information about seasonal flu is available. Already there have been a number of studies attempting to model the H1N1 pandemic (e.g. [6]–[10]). Intriguingly, despite the fact that our knowledge of seasonal influenza is at a relatively advanced level, few if any attempts have made use of this information to derive forecasts for the H1N1 pandemic by simple extrapolation. Even basic back-of-the-envelope calculations are lacking. We follow this path by first using the known characteristics of seasonal influenza epidemics to estimate the basic parameters 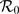 and 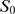 which fully determine the epidemic dynamics in the context of the well known SIR model. This includes taking into account the duration of the outbreak, an important factor that is often neglected.

The standard approach for fitting epidemic models is to use detailed data from epidemic curves as obtained through surveillance. However, we believe that the type of rough fitting proposed here is useful as a complement, with the advantage that it depends only on robust characteristics of influenza epidemics and is thus less sensitive to the uncertainties involved in the surveillance process. Furthermore, we think it is useful for epidemiologists to be able to make simple calculations such as those demonstrated here, which can serve as a check on results obtained by more computationally intensive fitting methods (e.g. MCMC methods), and also help develop the modeler's “feel” for the processes and quantities involved.

Having estimated the key parameters 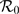, 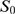 for seasonal influenza, we then proceed to make projections for the expected attack rate of pandemic influenza, under the assumption that pandemic influenza differs from seasonal influenza mainly in terms of the larger initial percentage of susceptibles in the population (although, as we argue, in contrast to many other investigators, taking the percentage of susceptibles to be 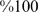 is questionable). The task is then to estimate the effect of the larger pool of susceptibles on the size of the epidemic, which we again approach by using results derived from the SIR model.

## Methods

To begin we recall that the SIR model assumes that the dynamics of Susceptible (S) and Infected (I) and recovered (R) fractions of the population are governed by the following equations [11]:
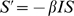
(1)

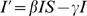
(2)

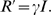
(3)


The contact rate between individuals is set at a constant 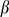, while the recovery rate is defined by the parameter 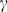.

In order to make predictions we need to fit the above model to epidemic incidence data for seasonal influenza. Three characteristic properties specific to seasonal influenza are drawn upon:

- The attack rate, 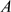, or fraction of a region's population infected over the entire influenza season, lies somewhere between 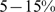 [12]. For convenience, the attack rate is approximated as 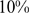, or 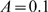.
- The duration of seasonal influenza epidemics is approximately 3 months [13].
- The average infectious period of a sick individual is 3 days [14]. Since, by (2), the average length of time that an individual spends in the 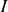 compartment is 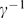 days, this implies that 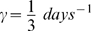.

We now show that these three properties are sufficient to fit the SIR model.

Since 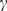 is determined by (c), fitting the SIR model requires determining 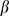 and the initial condition 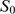. It is assumed that the initial number of infectives is nearly negligible.

A key epidemiological parameter is the basic reproduction number [15]

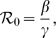
which denotes the number of individuals infected by a single infected individual placed in a totally susceptible population. (Of course estimating 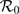 immediately gives us an estimate of 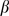). At least as important is the effective reproduction number (see eg., [3], [16])
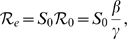
(4)which denotes the number of individuals infected by a single individual placed in a population with fraction 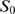 of susceptibles.

To proceed further it is necessary to take into account the epidemic's duration.

For our purposes the duration of an epidemic is defined as the length 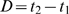 of the time period 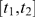 such that 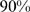 of infections occur during this period, along with the condition 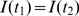. For the SIR model this duration can be expressed in an integral formula (see Supporting Text S1) which depends only on 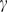 and on 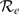. This integral may be computed for various values of 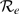 and 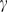, and typical results are displayed in table 1. This enables us, assuming that 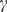 and the duration of an epidemic is known, to determine the value of 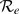 for which the duration of the epidemic fits the one predicted by the SIR model.

**Table 1:** Duration of an epidemic (in days) as a function of 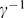 (average infection period) and 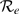.

	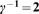	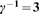	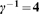	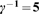
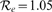	239.5	359.1	479.0	599.0
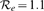	121.7	182.4	243.3	304.2
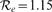	82.4	123.6	164.8	206.0
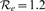	62.7	94.1	125.5	156.9
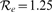	50.9	76.4	101.9	127.3
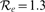	43.1	64.7	86.2	107.7
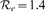	33.2	49.9	66.5	83.1
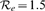	27.3	41.0	54.7	68.4

Once 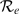 is chosen appropriately it is possible to determine the population's initial susceptibility 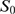 as follows. First, we need 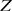, the fraction of susceptibles who are infected during the epidemic, which can be found as the solution of the final-size equation [11], [17] (see also Supporting Text S1):
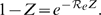
(5)


Since the attack rate is known ((a) above) and is given by 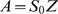, we estimate the fraction of susceptibles in the population as 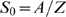.

## Results

### Fitting parameters for seasonal influenza

Since, by (b), seasonal flu lasts approximately 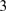 months, table 1 shows that, assuming 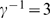 days, it becomes necessary to take 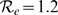 to fit the duration of the epidemic.

Substituting 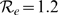 in (5) and solving numerically, we obtain 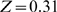, that is 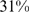 of the susceptibles become infected. Therefore the attack rate is 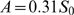. Based on the assumption that the attack rate for seasonal flu is 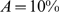, we conclude that 
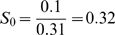
. That is, at the beginning of the season some one third of the population is susceptible and has the potential to be infected. This level of susceptibility seems reasonable given that a large component of the population has most likely gained immunity from previous exposure to related strains of the current influenza virus. Finally, since 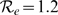 and 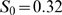, we obtain 
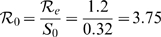


### Predicting attack rate for pandemic influenza

Having estimated parameters for seasonal influenza, consider now the arrival of pandemic influenza into a region. In the absence of previous exposure to the pandemic, it is reasonable to assume that a much larger proportion of the population is susceptible than is the case for the seasonal flu. Our working hypothesis is that there are no other relevant epidemiological differences between the two types of influenza. As far as the average duration of infectivity (or, equivalently, the mean serial interval), 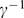, there is some direct evidence based on infection networks that it is indeed close to that of seasonal influenza [18], [19]. We shall henceforth assume (in the absence of hard data) that the intrinsic transmissibility 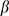 is also the same. Therefore we are assuming, in particular that 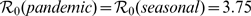. It should be noted that this assumption disregards the effect of seasonality on the reproduction number: our estimate 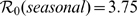 is appropriate for the winter season, in which seasonal flu occurs. Therefore our working assumption that 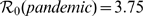 is most appropriate when the pandemic virus is introduced into the population at a date close to the usual initiation period of the seasonal influenza epidemic. This was the case for the current H1N1 pandemic in the southern hemisphere, and less so for the northern hemisphere, where the virus was introduced ‘out of season’. However, since quantitative knowledge about the extent of the effect of seasonality on the transmission rate is meager, we do not take this factor into account in the following estimates.

When modeling pandemic influenza and estimating 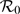 it is often taken for granted that the entire population is susceptible. This assumption, however, has been shown to be questionable (Mathews et. al. 2007, McCaw et. al. 2009). Indeed Mathews et al. (personal communication), based on model-fitting, have estimated that in the 1918 pandemic in the UK only 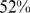 (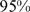 confidence interval 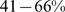) of the population were susceptible. Moreover, we argue that by considering the expected duration of an epidemic, calculations based on the SIR model indicate that 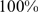 population susceptibility is unlikely: it would imply 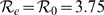, which would lead to an epidemic with an extremely short duration of 12 days. Examining epidemic curves from past pandemics indicates that their duration is indeed shorter than those of seasonal influenza epidemics, but not to such an extent, and usually of the order of one month. (Note, however, that small isolated communities such as documented in Alaska in 1918 exhibited 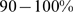 attack rates [1], and thus provide examples where 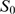 may be as large as 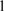). Lastly, let us note that the various current estimates of the effective reproductive number [2], [7], [10], [20], [21] for the 2009 H1N1 influenza give results in the range 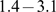. It is important to note that as most of these estimates are based on the initial growth rate of the epidemic, these are actually estimatesof 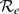 and *not* of 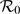 [3]. Under our assumption 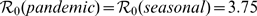, it follows that 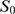 must be significantly less than 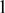 in order for 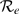 to be in the range of these previous estimates.

In the following we begin by assuming there is 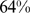 susceptibility for pandemic influenza (that is, since the number of infected at the beginning of the epidemic is very small, we assume 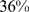 of the population is initially immune), which is twice that we estimated for seasonal influenza. How then does this simple difference in population susceptibility change the influenza attack rate? A naive approach might suggest that if there are twice as many more susceptibles in the population, the attack rate for the pandemic might be expected to be 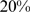 of the population instead of 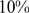 for seasonal influenza.

The appropriate calculation of the attack rate (as based on the SIR model) involves two stages:

- Determining the fraction 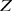 of susceptibles who become infected during an epidemic as the solution of the final-size equation (5).
- Calculating the attack rate as given by 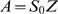.

Since we assume that 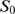 is twice as high for the pandemic influenza as for the seasonal flu, we obtain that 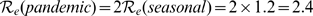. From (i) & (ii) above, a larger value of 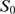 increases the size of the epidemic in *two* ways. Firstly, the quantity 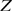, the fraction of susceptibles who become infected, is much larger. Solving (5) for 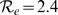 we obtain 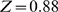 (that is - 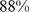 of susceptibles will be infected during the pandemic), in contrast to 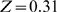 for seasonal flu. Secondly, there are more susceptibles so that the attack rate 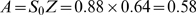. Thus although the estimated number of susceptibles for the pandemic is twice that for the seasonal flu, the resulting attack rate is 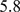 times higher (and 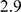 higher than the “naive” prediction).

## Discussion

**Figure 1:**
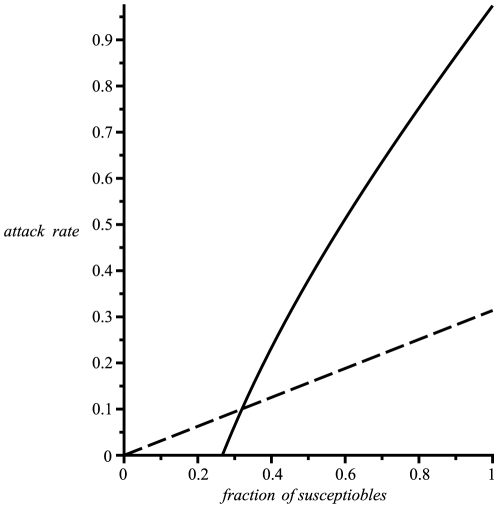
Attack rate as a function of the initial fraction of susceptibles. Assuming 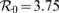, the attack rate (continuous line) is plotted as a function of the initial fraction of susceptibles 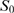 in the population. An epidemic will not trigger unless the initial susceptibles are greater than 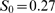, due to herd immunity. The dashed line shows the naive prediction for the attack rate, obtained by extrapolating linearly from the 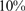 attack rate for 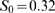, which can be well below the theoretical estimate.

It is interesting to note that the naive prediction is based on the supposition that doubling the number of susceptibles should double the number of people infected. However the flaw in this logic derives from the collective phenomenon whereby for low levels of susceptibles the population inherits a protection akin to herd immunity [15]. That is, large numbers of immune individuals tend to block infection routes and thereby reduce the risks of infection for the entire population. Thus increasing the number of susceptibles leads to a breakdown in herd immunity and effectively amplifies the risks of the epidemic to levels well beyond the naive prediction. This is demonstrated in Figure 1 which displays a graph of the true attack rate as a function of 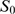 and provides a comparison with the naive prediction.

It should be stressed that the above estimates are subject to the uncertainty in the estimate of the attack rate of seasonal influenza (assumption (a)) as well as to uncertainty in the actual fraction of the population susceptible to the pandemic influenza (which we took to be 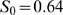). Nevertheless, the goal here is not to give exact predictions but to convey the conceptual mechanisms involved, and sometimes the nonintuitive outcomes when a pandemic triggers. Most significantly, the epidemic attack rate can reach unexpectedly high levels.

At this point in time the attack rate of the current H1N1 pandemic is not known with any confidence, and the results of serological studies which can shed light on this are awaited. Nevertheless, based on sureveillance data, it appears that while the outbreaks in various countries have been larger than those of seasonal influenza, they have not been as large as would be predicted based on the above estimates (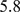 times as large as seasonal epidemics). If this indeed turns out to be the case, it becomes an important question for epidemiologists to explain this discrepancy. There are several possibilities which need to be considered:

1. It may be that the fraction of susceptibles is even lower that the value 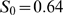 that we posited, which would result in reduction of the value of 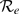. If this is the case, then the biological mechanisms behind a considerable prior immunity need to be investigated.
2. It may be that, contrary to what was posited above, the reproduction number 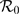 (hence also 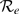) for pandemic H1N1 is considerably *lower* than that for seasonal influenza, reflecting a lower transmissibility. If this is true, it could be explained as a result of the fact that the seasonal strains, having already co-evolved with the human population's immunity, have developed higher transmissibility, in comparison with the new swine flu virus which has not yet had this opportunity.
3. While the above SIR modeling analysis provides an outline of the processes at work, it obviously does not take into account a number of subtleties and complexities characteristic to influenza dynamics in heterogeneous populations. It could be that some of these effects could lead to a reduction of the the relative sizes of pandemic influenza as compared to seasonal influenza, as predicted by the above analysis. If this is indeed the case, it is important for future modeling studies to identify what are these important factors that must to be taken into account.

We believe that the considerations and calculations presented here, and the questions raised, can serve as a starting point for stimulating future debate around these important issues.

## Supporting Information

Text S1 We derive characteristics of an epidemic described by the SIR model in terms of the parameters of the model.
(0.09 MB PDF)
