# Supplementary material for: Pandemic Dynamics and the Breakdown of Herd Immunity
Source: PLoS One. 2010 Mar 15;5(3):e9565. doi: 10.1371/journal.pone.0009565 (PMC2837721; doi:10.1371/journal.pone.0009565)
Supplement: Text S1 — We derive characteristics of an epidemic described by the SIR model in terms of the parameters of the model. (0.09 MB PDF) [file pone.0009565.s001.pdf]

# Supporting Text for "Pandemic dynamics and the breakdown of herd immunity"

Guy Katriel, Lewi Stone

## 1 Deriving characteristics of an epidemic from the SIR model

We derive analytic expressions for the attack rate $A$ and for the the duration $D$ of an epidemic described by the SIR model

$$S' = -\beta IS, \quad (1)$$

$$I' = \beta IS - \gamma I, \quad (2)$$

$$R' = \gamma I. \quad (3)$$

with the conditions

$$S(-\infty) = S_0, \quad I(-\infty) = 0, \quad R(-\infty) = 1 - S_0. \quad (4)$$

in terms of the parameters $\gamma$, $\mathcal{R}_0 = \frac{\beta}{\gamma}$, $\mathcal{R}_e = S_0\mathcal{R}_0$.

Since $A, D$, and $\gamma$ are approximately known (see assumptions (a)-(c) in the main text), these expressions enable us to estimate the parameters $\mathcal{R}_e, \mathcal{R}_0, S_0$.

### 1.1 The attack rate

The attack rate in the SIR model is given by the final size formula, which is well-known, see e.g. [1] and recalled for the reader's convenience. From (1),(15), we have so that

$$S(t) = S_0 e^{-\beta \int_{-\infty}^{t} I(s)ds}. \quad (5)$$

From (1),(2) we have

$$I'(t) + \gamma I(t) = -S'(t)$$

and integrating both sides, using (15), we have

$$I(t) + \gamma \int_{-\infty}^{t} I(s)ds = S_0 - S(t). \tag{6}$$

Substituting (6) into (5) we have

$$S(t) = S_0 e^{-\mathcal{R}_0[S_0 - S(t) - I(t)]}. \tag{7}$$

Taking $t \to \infty$ we have

$$S(\infty) = S_0 e^{-\mathcal{R}_0[S_0 - S(\infty)]},$$

and setting

$$Z = 1 - \frac{S(\infty)}{S_0}$$

we rewrite

$$1 - Z = e^{-\mathcal{R}_e Z}. \tag{8}$$

Note that $Z$ is the fraction of susceptibles who get infected during the epidemic. Note that this fraction is determined by $\mathcal{R}_e$ (we mention that, in the notation of [1], $R_0$ is what we here call $\mathcal{R}_e$). The attack rate (the fraction of the population who get infected during the epidemic) is

$$A = S_0 Z. \tag{9}$$

Note also that, for given $\mathcal{R}_0$, the fraction $S_0$ of susceptibles affects the attack rate $A$ in two ways: first through its effect on $\mathcal{R}_e$ and hence on $Z$, and secondly through the relation (9).

We can also write (8) in terms of $A$, as

$$1 - \frac{A}{S_0} = e^{-\mathcal{R}_0 A}. \tag{10}$$

## 1.2 Duration of the epidemic

We must give a precise definition of what we mean by the "duration of the epidemic". We define the epidemic period $[t_1, t_2]$ by the following conditions

(1) %90 of the cases occur within this period, that is $S(t_1) - S(t_2) = 0.9A$,

(2) $I(t_1) = I(t_2)$.

The value $D = t_2 - t_1$ is called the duration of the epidemic.

There is some arbitrariness in the above definition. The value %90 can of course be replaced by a different fraction. In fact to be general we shall replace 0.9 by $\alpha$ in the derivations below, so that we have

$$S(t_1) - S(t_2) = \alpha A. \tag{11}$$

The condition $I(t_1) = I(t_2)$ could be replaced by the condition that the interval $[t_1, t_2]$ is centered at the peak of the epidemic (where the peak can be defined in at least two ways: as the time of the maximum of $I(t)$, or as the time of the maximum of the incidence $i(t) = -S'(t)$). This would not change the duration by much in practice, and is less convenient analytically, and we therefore chose the definition above.

**Lemma 1.** *The duration of an epidemic is given by*

$$D = \frac{1}{\gamma}\int_{\frac{\alpha Z}{(1-Z)^{-\alpha}-1}}^{\frac{\alpha Z}{1-(1-Z)^{\alpha}}} \frac{1}{u}\frac{du}{\mathcal{R}_e(1-u)+\log(u)}, \tag{12}$$

*where $Z$ is defined by (8).*

An important consequence is that the duration of an epidemic depends only on $\gamma$ and on $\mathcal{R}_e$.

*Proof.* From (7), and using $I(t_1) = I(t_2)$ and (11) we have

$$\frac{S(t_2)}{S(t_1)} = e^{-\mathcal{R}_0[S(t_1)-S(t_2)+I(t_2)-I(t_1)]} = e^{-\mathcal{R}_0[S(t_1)-S(t_2)]} = e^{-\alpha\mathcal{R}_0 A} \tag{13}$$

(11) and (13) provide us with two equations for $S(t_1), S(t_2)$ which can be solved to give

$$S(t_1) = \frac{\alpha A}{1-e^{-\alpha\mathcal{R}_0 A}}, \quad S(t_2) = \frac{\alpha A}{e^{\alpha\mathcal{R}_0 A}-1},$$

and using (10) we can rewrite this as

$$S(t_1) = \frac{\alpha A}{1-(1-\frac{A}{S_0})^{\alpha}}, \quad S(t_2) = \frac{\alpha A}{(1-\frac{A}{S_0})^{-\alpha}-1}. \tag{14}$$

Solving (2) for $I$ we have, fixing an arbitrary $t_0$,

$$I(t) = I(t_0)e^{-\gamma(t-t_0)}e^{\beta\int_{t_0}^{t} S(r)dr}.$$

Setting

$$x(t) = \log(S(t))$$

We have

$$x'(t) = \frac{S'(t)}{S(t)} = -\beta I(t) = -\beta I(t_0)e^{-\gamma(t-t_0)}e^{\beta\int_{t_0}^{t} S(r)dr}$$

and

$$x(t_0) = \log(S(t_0)), \quad x'(t_0) = -\beta I(t_0). \tag{15}$$

Therefore

$$x''(t) = \gamma\beta I(t_0)e^{-\gamma(t-t_0)}e^{\beta\int_{t_0}^{t} S(r)dr} - \beta^2 I(t_0)e^{-\gamma(t-t_0)}S(t)e^{\beta\int_{t_0}^{t} S(r)dr}$$

$$= -\gamma x'(t) + \beta e^{x(t)}x'(t)$$

so

$$x'(t) = \beta e^{x(t)} - \gamma x(t) + C$$

where, using (15),

$$C = \gamma\log(S(t_0)) - \beta(I(t_0) + S(t_0)),$$

so

$$x'(t) = \beta e^{x(t)} - \gamma x(t) + \gamma\log(S(t_0)) - \beta(I(t_0) + S(t_0)).$$

We note that $x(t)$ is independent of the choice of $t_0$, which shows that the expression $\gamma\log(S(t_0)) - \beta(I(t_0)+S(t_0))$ is independent of $t_0$, and in particular we may send $t_0 \to -\infty$ and obtain

$$x'(t) = \beta e^{x(t)} - \gamma x(t) + \gamma\log(S_0) - \beta S_0$$

$$= \gamma[\mathcal{R}_0 e^{x(t)} - x(t) + \log(S_0) - \mathcal{R}_0 S_0].$$

Thus

$$\frac{1}{\gamma}\frac{x'(t)}{\mathcal{R}_0 e^{x(t)} - x(t) + \log(S_0) - \mathcal{R}_0 S_0} = 1,$$

and integrating from $t_1$ to $t_2$ we have

$$\frac{1}{\gamma}\int_{t_1}^{t_2} \frac{x'(t)dt}{\mathcal{R}_0 e^{x(t)} - x(t) + \log(S_0) - \mathcal{R}_0 S_0} = t_2 - t_1.$$

Making the substitution

$$S = e^{x(t)}, \;\; dS = e^{x(t)} x'(t) dt$$

we get

$$\frac{1}{\gamma}\int_{S(t_1)}^{S(t_2)} \frac{1}{S}\frac{dS}{\log(\frac{S_0}{S}) - \mathcal{R}_0(S_0 - S)} = t_2 - t_1,$$

or, using (14),

$$D = t_2 - t_1 = \frac{1}{\gamma}\int_{\frac{\alpha A}{1-(1-\frac{A}{S_0})^{\alpha}}}^{\frac{\alpha A}{(1-\frac{A}{S_0})^{-\alpha}-1}} \frac{1}{S}\frac{dS}{\log(\frac{S_0}{S}) - \mathcal{R}_0(S_0 - S)}.$$

Finally, making the substitution $S = S_0 u$ gives (12). □

Using (12) we can calculate the duration of epidemics by numerical evaluation of the integral, with some results given in Table 1.

# References


[1] Murray J.D. Mathematical Biology 1989 Springer-Verlag New York, pp767.
